# Supplementary material for: Physicochemical properties of the vacuolar membrane and cellular factors determine formation of vacuolar invaginations
Source: Sci Rep. 2023 Sep 27;13:16187. doi: 10.1038/s41598-023-43232-5 (PMC10533490; doi:10.1038/s41598-023-43232-5)
Supplement: Supplementary file 1 — Supplementary Information. [file 41598_2023_43232_MOESM1_ESM.pdf]

Supplementary figures and tables

Physicochemical properties of the vacuolar membrane and cellular factors determine formation of vacuolar invaginations

Yoko Kimura[1,3], Takuma Tsuji[2], Yosuke Shimizu[1], Yuki Watanabe[1], Masafumi Kimura[1], Toyoshi Fujimoto[2], and Miyuki Higuchi[3]

[1]Graduate School of Integrated Science and Technology, Shizuoka University, Shizuoka, 422-8529, Japan,
[2]Laboratory of Molecular Cell Biology, Research Institute for Diseases of Old Age, Juntendo University Graduate School of Medicine, Tokyo, Japan
[3]Department of Agriculture, Shizuoka University, Shizuoka, 422-8529, Japan

Fig. S1

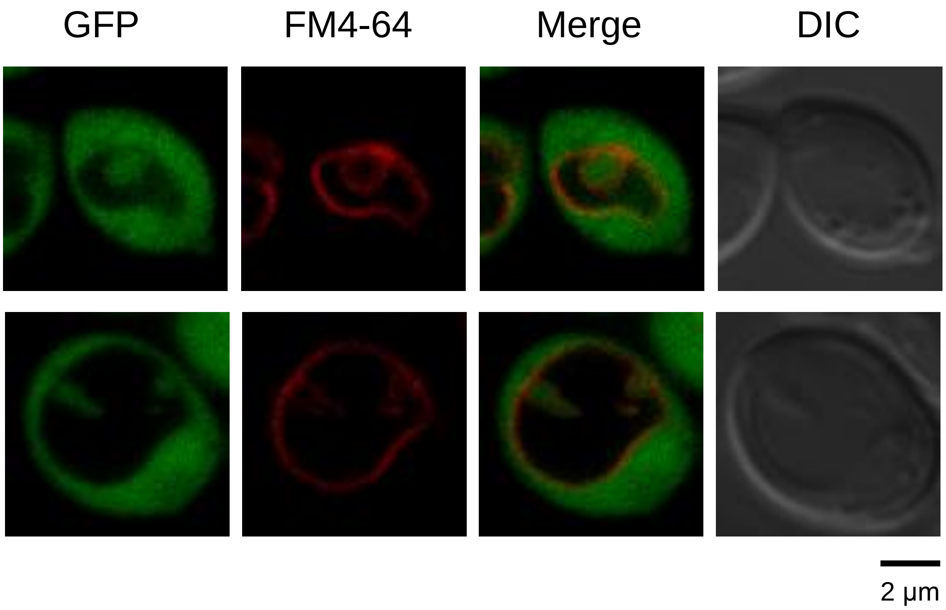

Fig. S1 Invaginated vacuoles of *hfl1*Δ cells expressing Pgk1-GFP grown at 25° C.

Fig. S2

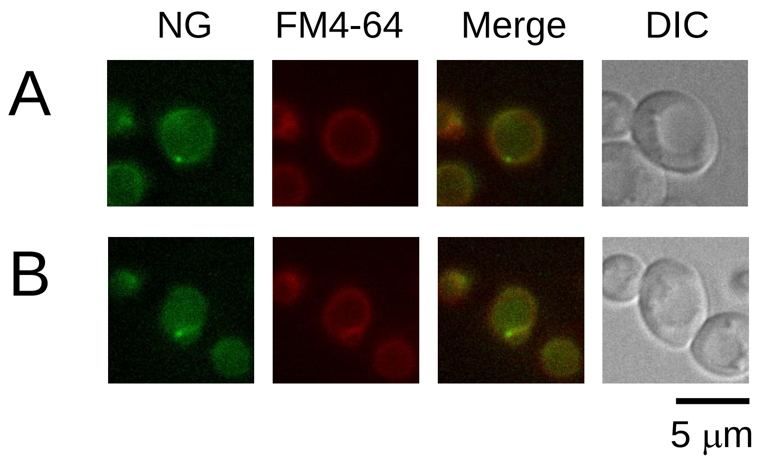

Fig. S2 Images of Hfl1-NG foci of cells grown at 25° C

At 25° C, Hfl1-NG foci were mainly localized to the smooth vacuolar membrane. Two cells are shown.

Fig. S3

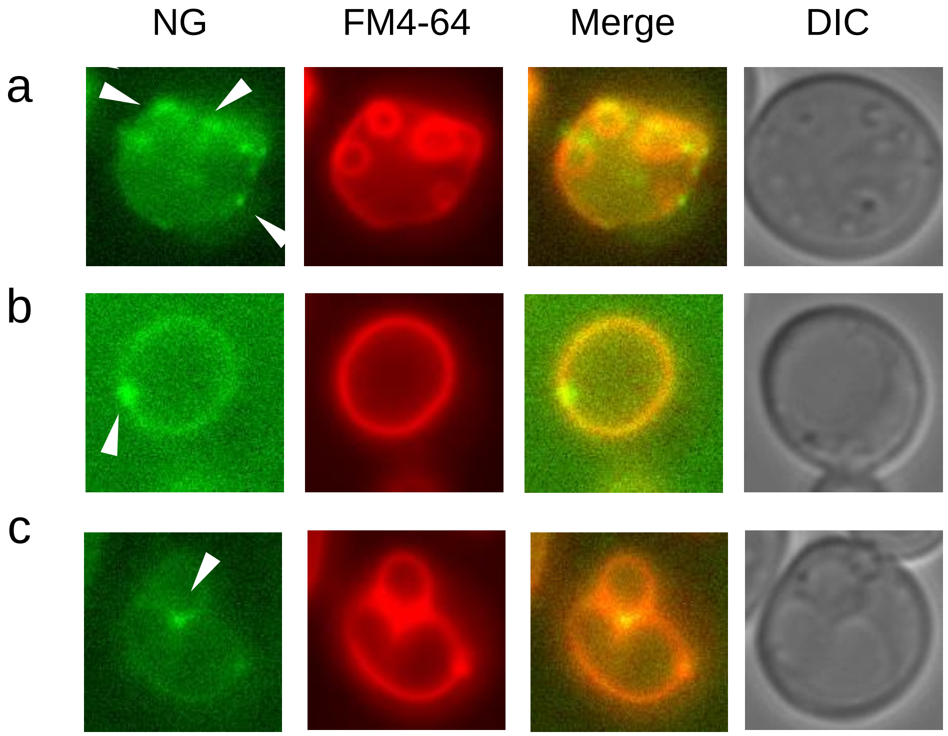

Fig. S3 Different localizations of Hfl1-NG foci.

Examples of Hfl1-NG foci are shown. Foci are indicated by arrowheads. Image taken from a cell after heat stress for 4 h.

(a) Type A. Hfl1-NG foci at the neck of the invagination.

(b) Type B. Hfl1-NG foci on the smooth membrane.

(c) Type C. Hfl1-NG at the contact site of two vacuoles.

Fig. S4

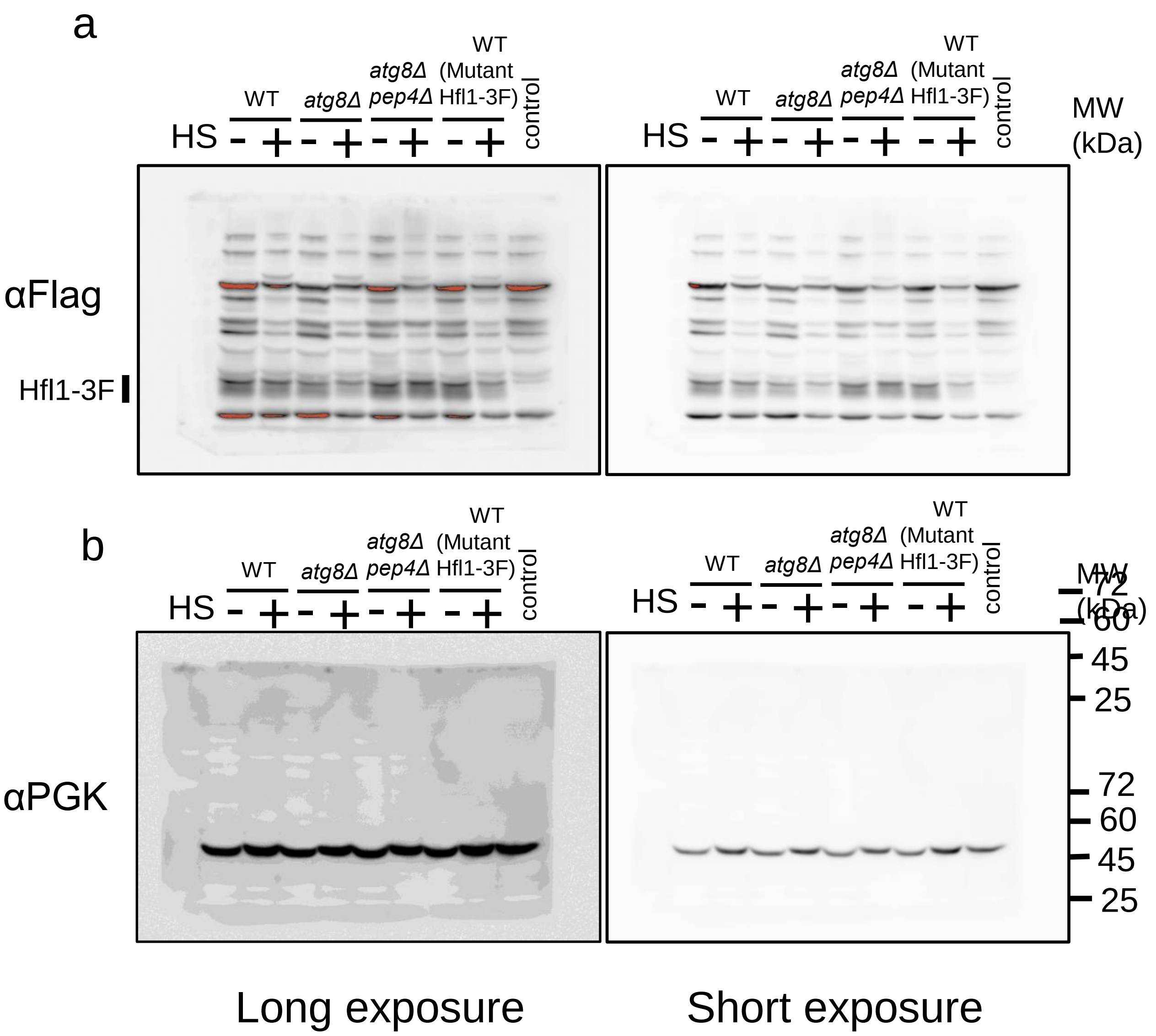

Fig. S4 Blot transparency

Uncropped images of the blots shown in Fig.5.

(a) Anti-Flag antibody. Overexposed signals are reddened.

(b) Anti-Pgk1 antibody.

Fig. S5

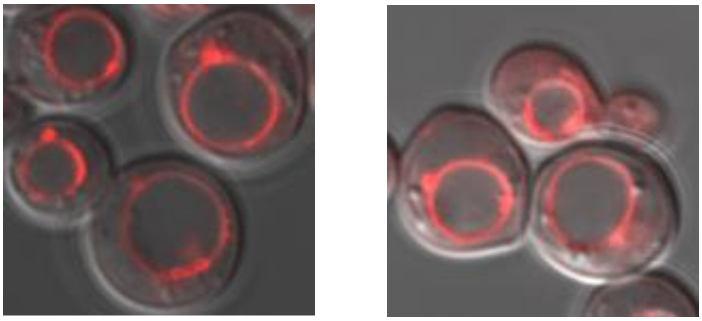

hfl1Δvps23Δ *hfl1Δvps4Δ*

Fig. S5 Vacuolar morphologies of *hfl1Δ vps23Δ* and *hfl1Δvps4* cells grown at 40.5° C for 4 h

Merged images of FM4-64 fluorescence and DIC.

Fig. S6

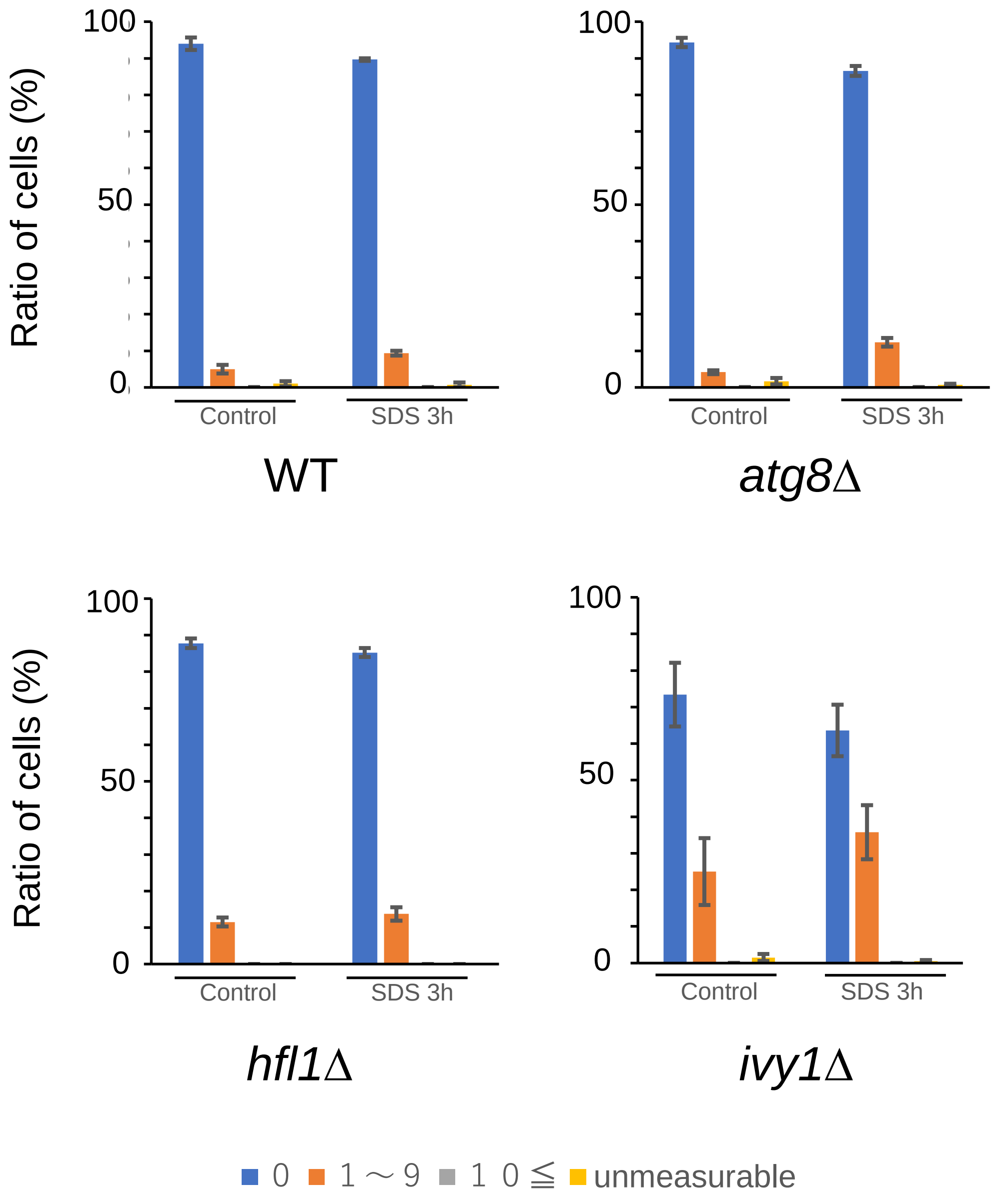

Fig. S6. Quantification of vacuolar invaginations of WT, *atg8*Δ, *hfl1*Δ, and *ivy1*Δ cells with or without SDS treatment.
The experiment was repeated three times and the mean ± SE is shown.

Table S1 Strains used in this study

Name	Genotype	additional phenotype	Cross/Source/Reference
W303a	MATa *ade2-1 can1-100 his3-12,16 leu2-3,112 trp1-1 ura3-1*		Rothstein
W303α	MATα *ade2-1 can1-100 his3-12,16 leu2-3,112 trp1-1 ura3-1*		Rothstein
Y1579	W303a, *hfl1Δ::KanMX*		this study
Y1623	W303α, *hfl1Δ::KanMX*		this study
Y1373	W303a, *atg8Δ::KanMX*		Ishii et al, 2019
Y1493	W303α, *atg8Δ::KanMX*		Ishii et al, 2019
Y1375	W303a, *ivy1Δ::KanMX*		Ishii et al, 2018
Y1751	W303a, Zrc1-GFP::HIS3		this study
Y1760	W303a, Ybt1-GFP::HIS3		this study
Y1633	W303a, *bar1, Vph1-GFP::HIS3MX*		Ushimaru
Y1891	W303a, *bar1, Vph1-GFP::HIS3MX, atg8Δ::KanMX*		this study
Y1637	W303a, *bar1, Vph1-GFP::HIS3MX, hfl1Δ::KanMX*		this study
Y1390	W303a *PGK1-GFP::HIS3*		Ishii et al, 2019
Y1632	W303a *PGK1-GFP::HIS3, hfl1*Δ::KanMX		this study
Y1412	W303a *PGK1-GFP::HIS3, atg8*Δ::KanMX		Ishii et al, 2019
Y1287	W303a, *vps24Δ::KanMX*		Ishii et al, 2018
Y1286	W303a, *vps4Δ::KanMX*		this study
Y1406	W303a, *atg8Δ::KanMX, vps24Δ::KanMX*		Ishii et al, 2019
Y1721	W303a, *hfl1Δ::KanMX, vps24Δ::KanMX*		Y1287 x Y1623
Y1724	W303a, *hfl1Δ::KanMX, vps4Δ::KanMX*		Y1286 x Y1623
Y1539	W303a, *atg8Δ::KanMX , ivy1Δ::KanMX*		Ishii et al, 2019
Y1542	W303a, *atg8Δ::KanMX , ivy1Δ::KanMX, PGK1-GFP::HIS3*		Ishii et al, 2019
Y1700	W303a, *hfl1Δ::KanMX , ivy1Δ::KanMX*		Y1375 x Y1623
Y1711	W303a, *hfl1Δ::KanMX , ivy1Δ::KanMX, PGK1-GFP::HIS3*		this study
Y1913	W303a, *hfl1Δ::KanMX , ivy1Δ::KanMX, VPH1-GFP::HIS3*		this study
BY20222	W303a, *RAD5*		NBRP
Y1430	W303a	Ade+	Sakai
Y1489	W303α, *ADE+*	Ade+	W303α Y1430
Y1508	W303a, *RAD5*	Ade+	Y1489 x BY20222
Y1797	W303a, HFL1-ymNeongreen::SpHIS5, *RAD5*	Ade+	this study
Y1881	W303a, HFL1-ymNeongreen::SpHIS5, *atg8*Δ::KanMX, *RAD5*	Ade+	this study
Y1807	W303a, HFL1-3xFlag::HIS3		this study
Y1811	W303a, HFL1-3xFlag::HIS3, *atg8*Δ::KanMX		this study
Y1870	W303a, HFL1-3xFlag::HIS3, *atg8*Δ::KanMX, *pep4*Δ::*HIS3*		this study
Y1860	W303a, HFL1-3xFlag::HIS3, *pep4*Δ::*HIS3*		Y553xY1807
Y553	W303α, *pep4::HIS3*		this study
Y1916	W303a, hfl1(W371A, I375A, D384A, Y387A)-3xFlag::*HIS3*		this study
BY4741	MATa *his3Δ1, leu2Δ0, met15Δ0, ura3Δ0*		Euroscarf
	BY4741, *hfl1*Δ::KanMX		Euroscarf
	BY4741, Zrc1-GFP::*HIS3*		Ushimaru
	BY4741, Ybt1-GFP::*HIS3*		Ushimaru
	BY4741, *vps4*Δ::KanMX		Euroscarf

Table S2 Plasmids used in this study

No.	Name	Source
E1068	pRS306-HFL1 (D384A, Y387A, W371A, I375A)	this study
E1059	pRS306-HFL1 (D384A, Y387A)	this study
E1055	pRS316-HFL1	this study
E1058	pRS306-HFL1	this study
V168	pFA6a-link-ymNeongreen-SpHis5	addgene.org/125704
V165	pBSII-3xFlag-TCyc1-HIS3	this study
V110	pBSII-3xFlag-TCyc1-KanMX6	Yashiroda, H.
V016	pRS313	Sikorski and Hieter, Genetics 122, 19-27, 1989
V107	pRS306	Sikorski and Hieter, Genetics 122, 19-27, 1989

Table S3 Oligonucleotides used in this study

No,	Name	Sequence
1219	VPS24 up350	CCTTAGAAAATACATTCCAATCCTACGAATAGACG
1220	vps24 down 390	CGGTATTGAGTCCATGTTGAAGCACTATGTGGATGAAGTG
1421	VPS4up370	CGTTAGTGACTTCAAGTCCGATTCACATGTCGCCACTC
1423	VPS4down430	CTGGGACTCCGACGCCGACTTCTATTCCTCATGAAG
1358	ATG8 down 410 AS	CTGTAAGAAATTGATTCTACATACATAAGAC
1360	Atg8 up391	GCTCAAAGACAGACCCAATTGGTGATGAAG
1370	PGK1 1570 down 370AS	GTACAATAGATGGTAAAGAATATTGGCAGCGC
1371	PGK1 745	CTGAAATCGGTGACTCCATCTTCGACAAGGC
2105	VPH1 3211	GGCCTTATCATTGGCACATGCTCAATTGTC
2107	VPH1 3990 as	ACGTTAATGGCCCATAGAGCACCCATACCG
1594	HFL1up455	CAAGTTTACCCATCTTTACCTACATAAGAG
1605	HFL1 down 276AS	GCCAAAATGCATTGGTGGTGGTGCTGTTGC
1706	HFL1-w	CAAATACACAGCATTTAGCATACCTTATGG
1707	HFL1-x	GGGGATCCGTCGACCTGCAGCGTACGAACAAGCCATTGATG ACCTACTTTGAACATCTC
1708	HFL1-y	GTTTAAACGAGCTCGAATTCATCGATGAGTCGTTAAATGTACG TATATACATAGTT
1709	HFL1-z	CATTTCTTGGACTGCATATGAATGTGCTAAG
1819	YBT1 4528	CAAGCAATGAGGCTTCGTCGACTAACTCCG
1820	YBT1 5454AS	CTGTCACTAAATTCATCATATGGGTCTAGG
1821	ZRC1 956	GGTCCATGACTTCCACGTCTGGAACTTAAC
1822	ZRC1 1815 as	GATCCGAGTAATATCTCCAAGAATATTCAG
1836	HIS3 EcoRI 303 in pRS313	AATTGAATTCTTGGCCTCCTCTAGTACACT
1837	HIS3 BamHI as 1276 in pRS313	AATTGGATCCTTGTCATCTTCAGTATCATA
1845	HFL1 oligo1 for 3Flag	TCAATGAATGATTTAAGAAGAGATGTTCAAAGTAGGTCATCAA TGGCTTGTGACTACAAAGACCATGACGG
1846	HFL1 oligo2 for 3Flag	TGGATTGCATAAAGACTGTATTACAGGTTAGAGGTAGCGAAA CTGTCTCCCCTTGTCATCTTCAGTATCATAC
1967	Hfl1 up760 SalI remake	AATTGTCGACAGTGGGTTTGTCATTGGGTT
1968	Hfl1 down375 EcoRI as	AATTGAATTCTTGGACTGCATATGAATGTG
1985	HFL1 D384A Y387A sense	GAACTTTTCCTGAGGCTCCAAATGCTCCGGTTGTTCACGACT AC
1986	HFL1 1145 as	GGAAAAGTTCTCTGACCAGCAATATCATCTTCCCATG
2099	HFL1 KpnI 3136 as	CGCACTGAGTGGTACCATTGCTACCATTGC
2101	HFL1 1708	CAAGATCGAACCTCTGGATATGTGTACAAG
2102	HFL1 2221 antisense	GCCATTGATGACCTACTTTGAACATCTCTT
2103	HFL1 2221 sense	AAGAGATGTTCAAAGTAGGTCATCAATGGC
2104	HFL1 2647 antisense	GCTAATATACCAGCCACTGCCATTTCTTGG

2114	HFL1 W371AI375A	CCGGAATCAATAGAGGAATCAGCGGAAGATGATGCTGCTGG TCAGAGAAC
2115	HFL1 1115 as	CTATTGATTCCGGAACAAGGTTGTTATCTATACTCCCATATG
